# Reconstructive Limb Salvage After COVID-19-Induced Gangrene and Amputation

**DOI:** 10.7759/cureus.60758

**Published:** 2024-05-21

**Authors:** Mark Swerdlow, Gavin T Kress, Laura Shin

**Affiliations:** 1 Vascular Surgery, Keck School of Medicine of USC (University of Southern California), Los Angeles, USA

**Keywords:** reconstruction, limb salvage, prothrombotic, covid toes, ischemia, covid-19

## Abstract

This case series describes the clinical course and reconstructive methods utilized for patients with diabetes and significant gangrene and necrosis following coronavirus disease 2019 (COVID-19) infection. COVID-19 produces mainly respiratory symptoms but has a variety of atypical presentations and sequelae. Serious complications are increased in patients with underlying medical conditions such as diabetes mellitus. By generating a prothrombotic milieu, severe acute respiratory syndrome coronavirus 2 (SARS-CoV-2) increases the risk for arterial and venous thromboses. Inflammatory damage and micro-thromboses are thought to contribute to acro-ischemia, colloquially known as ‘COVID toes,’ which presents cutaneously as chilblain-like lesions. Necrosis can be severe and devastating, often resulting in major amputation. Two exemplary case reports are presented herein: first, a 57-year-old female presented for vascular evaluation with pedal gangrene to the midfoot one month after developing painful discoloration in her right toe. After angioplasty restored pedal blood flow, she received a transmetatarsal amputation (TMA) with a local tissue flap. Second, a 41-year-old female presented for vascular evaluation with extensive pedal gangrene three months after hospitalization for COVID-19. After arteriotomy improved pedal blood flow, she underwent a Lisfranc amputation followed by superficial circumflex iliac artery perforator (SCIP) flap reconstruction. Sufficient evidence suggests that COVID-19 impairs microcirculatory function and can be especially detrimental in diabetic patients. Reconstructive techniques in patients with severe gangrene with COVID toes help patients regain functionality.

## Introduction

Coronavirus disease 2019 (COVID-19), caused by a newly emergent severe acute respiratory syndrome coronavirus 2 (SARS-CoV-2), produces mainly respiratory symptoms but has a variety of atypical presentations and sequelae [1]. Serious complications are increased in patients with underlying medical conditions such as diabetes, heart disease, lung disease, and cancer. By generating a prothrombotic milieu, SARS-CoV-2 increases the risk for arterial and venous thromboses, including myocardial infarction, stroke, and microvascular thrombosis [2]. This last condition is thought to contribute to acro-ischemia, colloquially known as ‘COVID toes,’ which presents cutaneously as chilblain-like lesions, although some believe it results from microvascular inflammatory damage without microthrombosis [3]. Here we present a case series of two patients at the Keck School of Medicine of USC, Los Angeles, USA, who suffered gangrene from COVID toes resulting in pedal amputations; one received pedal reconstruction with a local fillet flap (soft tissue rearrangement), and the other with a superficial circumflex iliac artery perforator (SCIP) flap. The informed consent was obtained from both patients for participation in this report.

This article was previously presented as an abstract at the American Diabetes Association 82nd Scientific Sessions in June 2022.

## Case presentation

Case 1 (local flap)

**Figure 1:**
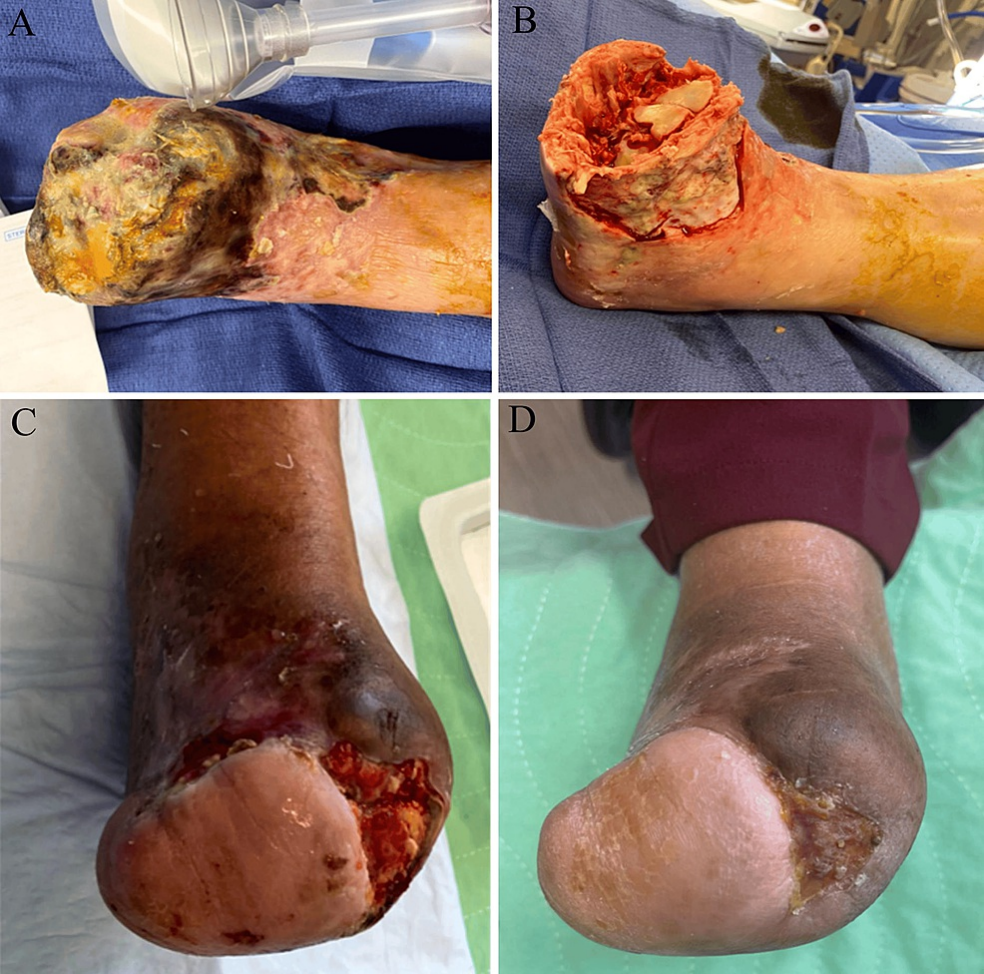
Case 1 progression showing: A) patient’s right foot on presentation; B) after debridement, one week post-TMA; C) seven weeks after local tissue rearrangement/local flap procedure; D) four months after local flap procedure. TMA: transmetatarsal amputation

A 57-year-old Hispanic, non-smoking female, with a medical history of hypertension, diabetes mellitus, end-stage renal disease on dialysis, and hypercholesterolemia, presented to the vascular clinic one month after first developing painful discoloration in her right toe during illness with COVID-19. She was evaluated by vascular surgery and podiatry for progressive gangrene and pain in her right foot. Doppler signals were absent bilaterally below the popliteal artery. Her right foot exhibited extensive gangrene of toes one through four and dorsal skin necrosis extending toward the lateral malleolus with tissue necrosis and tissue slough but without gross infection (Figure 1A). She was admitted for angiography, soft tissue cultures, antibiotics, and debridement [4]. A balloon angioplasty was performed two days after admission to restore blood flow to her anterior and posterior tibial arteries. She developed a right superficial femoral artery pseudoaneurysm, received a thrombin injection, and was discharged after an otherwise uneventful six-day hospital course. She was given prophylactic antibiotics for 14 days for positive soft tissue cultures. At her two-week follow-up visit, she was noted to have 1+ to 2+ palpable dorsalis pedis pulses and well-demarcated necrosis to her distal forefoot. One month after the initial presentation, she underwent open transmetatarsal amputation (TMA) and the application of a bilayered biologic graft. The patient returned to the clinic regularly for wound care and was noted to have a residual ankle contracture, with the ankle positioned at -15 degrees of plantarflexion (Figures 1B-1C). She had local wound care and negative pressure dressings for three months. During her next stage of reconstruction, she underwent debridement, local soft tissue rearrangement with the residual lateral flap, and percutaneous Achilles tendon lengthening. The closure of 70% of the stump was achieved and the medial area was debrided (Figure 1D). She had a small area of opening that was covered and began walking five months after her initial amputation. Her foot was completely healed, and she has been ambulating well with diabetic shoes and inserts without an assistive device.

Case 2 (SCIP flap)

**Figure 2:**
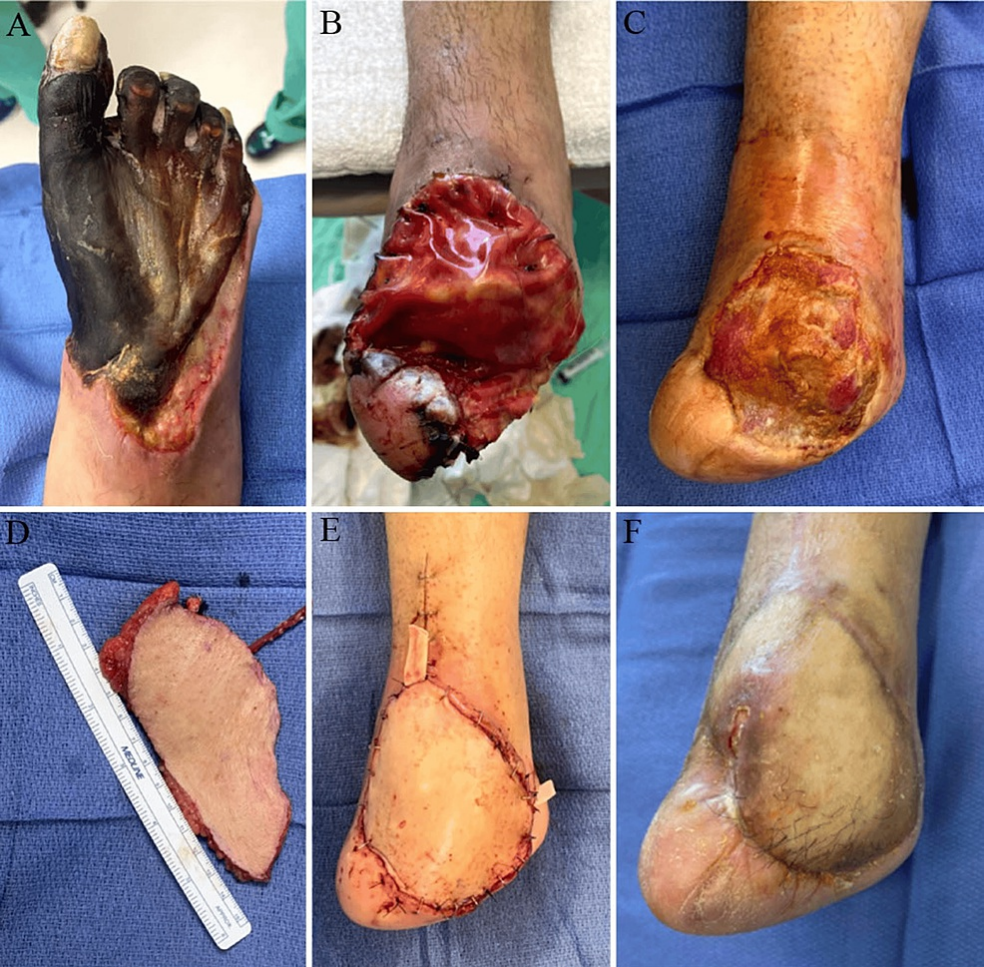
Case 2 progression showing: A) the patient’s right foot upon initial clinic visit; B) one week after Lisfranc amputation; C) immediately prior to the placement of the SCIP flap, 10 weeks after the amputation; D) the harvested SCIP flap; E) immediately following SCIP flap procedure; F) four months after SCIP flap procedure. SCIP: superficial circumflex iliac artery perforator

A 41-year-old Hispanic, non-smoking female with a significant medical history of type 2 diabetes mellitus presented to the vascular clinic with right foot gangrenous necrosis of all digits and tissue to the midfoot after COVID-19 infection (Figure 2A). She had a significant illness and underwent aortic stent placement, popliteal artery thromboembolectomy, and right lower extremity fasciotomies at an outside hospital three months after her infection. She had weakly palpable popliteal pulses bilaterally, monophasic Doppler-able right anterior and posterior tibial artery pulses, and absent distal pedal pulses. She was admitted for vascular studies to rule out residual thrombus from the popliteal embolectomy. She was admitted from the clinic and an arteriotomy between the below-knee popliteal artery and tibioperoneal trunk patched with a great saphenous vein graft restored pedal blood flow four days after admission. Immediately after her procedure, the pedal pulses were not palpable bilaterally, but her right posterior tibial pulse regained a Doppler signal; she was discharged after an uneventful three-day hospital course. At her one-week follow-up visit, the patient’s right foot displayed well-demarcated gangrenous tissue, and debridement of gangrenous tissue was performed. One week later, the patient underwent an open Lisfranc amputation with bilayered biological graft application and Achilles tendon lengthening. Her follow-up visits were at one, four, and seven weeks post-amputation, and her wound was granular and healing well, with no signs of infection or drainage (Figure 2B). Ten weeks after amputation, the patient underwent amputation revision with necrotic bone and tissue debridement and SCIP flap to improve tissue coverage and maintain length after the amputation (Figures 2C-2E). She was discharged after an uneventful four-day hospital course post-flap. Starting at one week postoperatively, she was followed in the clinic weekly until week 7, displaying a perfused and well-incorporated flap. She developed a small posterior ulceration at week 11 at the proximal aspect. During her ultrasound, a popliteal artery step-up was noted indicating a mild stenosis in the distal arteries. She underwent local wound care with protective antimicrobial dressings and subsequently underwent a repeat popliteal and peroneal artery angioplasty and ballooning. Pedal pulses were biphasic two weeks after her angioplasty, and her flap healed to completion (Figure 2F). She has been ambulating in a high-top shoe with a diabetic insert and filler, without an assistive device, and has returned to normal activity.

## Discussion

The incidence and pathophysiology of COVID toes remain largely unclear. The link between COVID-19 infection and cutaneous chilblain-like lesions seen in patients is not well defined and further studies examining the sequelae of COVID-19 are critical, especially in our high-risk patient populations. One study examining skin biopsies from seven adolescent patients with chilblain-like lesions demonstrated varying degrees of lymphocytic vasculitis and coronavirus particles in endothelial cytoplasm [5]. Another study analyzing 40 patients who presented with chilblain-like lesions during the COVID-19 pandemic found clinical, biologic, and histologic findings suggestive of a virus-induced type I interferonopathy but could not prove a causative link with SARS-CoV-2 [6]. Lastly, a study of 780 patients presenting with chilblain-like lesions before and during the pandemic found a weak correlation with COVID-19 infection and concluded the increased incidence of acral lesions may be due to pandemic-related behavior changes [7].

Multiple mechanisms have been proposed as an explanation for COVID toes; these processes are not mutually exclusive. Several studies have shown that SARS-CoV-2 patients admitted with other thromboses have positive antiphospholipid antibody markers, known to trigger endothelial dysfunction and platelet aggregation, in the absence of underlying coagulopathic or vasculitic conditions, suggesting an underlying virally-mediated antiphospholipid antibody syndrome [8]. Further, SARS-CoV-2 viral particles may directly damage endothelial cells or facilitate endotheliitis [5,9]. Additionally, SARS-CoV-2 can increase pro-inflammatory cytokines, including interleukin (IL)-2, IL-6, IL-7, and tumor necrosis factor-alpha (TNF-a), to levels not typically seen in bacterial sepsis or influenza and contribute to thrombosis formation via multiple mechanisms [2,3]. These theories may explain why microvascular thromboses develop in organs where coronavirus was not detected [2,10]. All of these theories, individually or in combination, could explain the development and increased incidence of chilblain-like lesions known as COVID toes.

## Conclusions

There's still much we don't know about the COVID toes phenomena, but sufficient evidence suggests that COVID-19 impairs microcirculatory function. We report the cases of two high-risk patients who underwent pedal amputation and reconstruction after recovering from COVID-19 and developing chilblain-like lesions. Anticoagulation therapy may be able to alter the course of microvascular thrombosis in COVID-19 patients.

## References

[1] Mallhi TH, Safdar A, Butt MH (2024). Atypical complications during the course of COVID-19: a comprehensive review. Medicina (Kaunas).

[2] Abou-Ismail MY, Diamond A, Kapoor S, Arafah Y, Nayak L (2020). The hypercoagulable state in COVID-19: incidence, pathophysiology, and management. Thromb Res.

[3] Hanff TC, Mohareb AM, Giri J, Cohen JB, Chirinos JA (2020). Thrombosis in COVID-19. Am J Hematol.

[4] Sharp CS, Bessman AN, Wagner FW, Garland D (1978). Microbiology of deep tissue in diabetic gangrene. Diabetes Care.

[5] Colmenero I, Santonja C, Alonso-Riaño M (2020). SARS-CoV-2 endothelial infection causes COVID-19 chilblains: histopathological, immunohistochemical and ultrastructural study of seven paediatric cases. Br J Dermatol.

[6] Hubiche T, Cardot-Leccia N, Le Duff F (2021). Clinical, laboratory, and interferon-alpha response characteristics of patients with chilblain-like lesions during the COVID-19 pandemic. JAMA Dermatol.

[7] McCleskey PE, Zimmerman B, Lieberman A (2021). Epidemiologic analysis of chilblains cohorts before and during the COVID-19 pandemic. JAMA Dermatol.

[8] Singh S, Zuwasti U, Haas C (2020). Coronavirus-associated coagulopathy: lessons from SARS-CoV1 and MERS-CoV for the current SARS-CoV2 pandemic. Cureus.

[9] Oudkerk M, Büller HR, Kuijpers D (2020). Diagnosis, prevention, and treatment of thromboembolic complications in COVID-19: report of the National Institute for Public Health of the Netherlands. Radiology.

[10] Rahi MS, Parekh J, Pednekar P, Mudgal M, Jindal V, Gunasekaran K (2023). Role of therapeutic anticoagulation in COVID-19: the current situation. Hematol Rep.

